# Editorial: Policy and regulation in bioengineering and biotechnology

**DOI:** 10.3389/fbioe.2023.1337663

**Published:** 2023-11-27

**Authors:** Andrea Wilcks, Hector Quemada

**Affiliations:** ^1^ Faculty of Health and Medical Sciences, University of Copenhagen, Copenhagen, Denmark; ^2^ Retired, Kalamazoo, MI, United States

**Keywords:** bioengineering, biotechnology, policy and regulation, new genetic technologies, synthetic biology, genetically modified organism (GM0)

## Introduction

The field of bioengineering and biotechnology is evolving at an unprecedented pace, making it crucial for policymakers, legislators, and regulatory bodies to ensure safe, sustainable, and efficient advancements. This Research Topic of papers explores the dynamic landscape of policy, legislation, and regulatory guidelines within this domain, highlighting their instrumental roles in shaping the future. The authors of these papers collectively contribute to a more informed and proactive future for bioengineering and biotechnology regulations. By examining, evaluating, and proposing policies, they are paving the way for a more secure and productive global biotech landscape that can meet the challenges of tomorrow. The papers featured in this Research Topic serves a dual purpose: 1) to scrutinize policy-related Research Topic, offering actionable recommendations for legislation in various areas of bioengineering and biotechnology and 2) to underscore the imperative of harmonizing policies and regulations, preferably on a global scale.

Comprising eleven papers, this Research Topic includes six reviews, three original research papers, one perspective paper, and one hypothesis and theory paper submitted by authors from Africa, Europe, Latin America, and the United States.

## Advancing risk assessment and management

Several papers propose strategies for enhancing the current risk assessment of bioengineered microbes or plants. For instance, Godbold et al. advocate that the inclusion of annotated sequences of concern (SoC) should be included in the risk assessment together with FunSoCs (Functional sequences of concern) to enhance the evaluation of genetically modified microorganisms, with particular emphasis on dual use research. Mueller aligns with this line of thought, using the origin of SARS-CoV-2 as a starting point to explore biorisk gaps not covered by existing policies. This investigation is especially pertinent in the context of the rapidly expanding field of synthetic biology. These discussions are crucial, as they pave the way for comprehensive risk assessments that encompass a broad spectrum of potential hazards.

Additionally, Buyel highlights the need for a more nuanced approach when employing plants as molecular farming organisms. He emphasizes that toxic compounds can pose risks even in the absence of replication*.* Buyel also calls for the assessment of risks associated with the host system, including the presence of toxic secondary metabolites, and the chosen production approach. His comprehensive overview of plant-based production, with a focus on product safety, offers stakeholders actionable recommendations to navigate the complex landscape of bioengineering.

In the realm of agricultural products, two papers scrutinize the existing regulatory framework and propose improvement for regulatory assessment. Kuzma et al. evaluate the regulatory assessment of three food and agricultural biotechnology case studies in the United States. Their evaluation leads to several policy suggestions intended to bolster oversight processes and promote sustainable agrifood products that rely on novel genetic technologies (NGT). Koller and Cieslak delve into the world of unintended genetic changes in plants caused by NGT, shedding lights on the relevance of comprehensive molecular characterization and risk assessment. They underscore the significance of assessing both intended and unintended genetic changes as part of a thorough molecular characterization and risk assessment for NGT plants intended for environmental release or market authorization. Their insights pave the way for more thorough risk evaluations in this burgeoning field.

## New applications and regulatory policies

The development of appropriate regulatory policies is paramount when introducing new applications to be released into unmanaged environments. An illustrative example is the release of gene drive-modified mosquitoes designed to control vector-borne diseases, as described by James et al. Their review articulates the importance of considering requirements and data needed before launching new products. This includes an examination of manufacturing and delivery requirements.

## The need for harmonized regulations

The diversity of knowledge and regulatory frameworks across countries and regions pose challenges in the field of bioengineering and biotechnology. While the widely differing approaches to regulation have been an obstacle with respect to transgenic organisms, the problem continues when countries deal with gene editing and other new genetic technologies. Several papers in this Research Topic address this Research Topic and offer recommendations to overcome it. Zarate et al. examine agricultural gene editing regulation in nine Latin America and the Caribbean countries. Their findings reveal the positive reception of harmonized regimes throughout the region*.* The benefits of coordination are evident, demonstrating how streamlined regulations can facilitate the responsible growth of bioengineering and biotechnology.

Masehela and Barros underscore the importance of coordinated policy and regulatory guidelines across the African continent. They highlight the advancements and challenges faced by various African countries in the development and implementation of biosafety policies. They call for an organized and coordinated approach in the region, underpinned by political will and commitment, to facilitate open discussions among scientists, regulators, and policy makers.

Mungeyi et al. provide a detailed overview of Namibian biosafety regulations and the implications for food and feed importers. They advocate for the reduction of administrative burdens, improved dialogue between regulators and the industry, and an increased awareness of regulations for feed and food importers. In line with Masehela and Barros, they propose that Namibia could learn from other countries and regions with established processes, thereby accelerating their own regulatory framework development.

From the European Union (EU) da Silva and Blasimme present a systematic review highlighting the impact of regulatory incentives on the rapid growth of organ chip research. Their analysis showcased how the convergence of research efforts, funding, and regulatory incentives has shaped a robust knowledge ecosystem that places many European research institutions as key international players in the field of organ chip research*.* This serves as an excellent example of how regional cooperation can advance research and innovation.

## Addressing inequities in biotechnology capabilities

Trump et al. investigate how risk culture contributes to disparities in biotechnology capabilities and how this could influence global inequities. They reveal how early adoption of biotechnology and regulatory frameworks can shape the development and acceptance of biotechnological innovations. The concentration of power in a few early adopter nations may hinder global collaboration, impede knowledge sharing, and potentially create a fragmented and competitive global biotech landscape. These findings emphasize the importance of a balanced, collaborative approach to global biotechnology advancement.

